# Socioeconomic differences in utilization of public and private dental care in Finland: Register-based evidence on a population aged 25 and over

**DOI:** 10.1371/journal.pone.0255126

**Published:** 2021-08-04

**Authors:** Mikko Nurminen, Jenni Blomgren, Hennamari Mikkola

**Affiliations:** The Social Insurance Institution of Finland (Kela), Helsinki, Finland; University of Georgia, UNITED STATES

## Abstract

Dental care utilization is known to have a strong socioeconomic gradient, with lower socioeconomic groups utilizing less of these services despite having poorer dental health. However, less is known about the utilization of dental services in the population concurrently in the public and private sectors in different socioeconomic groups. Additionally, evidence on how different sectors contribute to the overall socioeconomic gradient in dental care utilization is scarce. This study examines visits and absence of visits to public and private dentists in the years 2017–2018 by education, occupational class and income. Comprehensive register data was collected from the total population aged 25 and over in the city of Oulu, Finland (N = 118,397). The data were analyzed with descriptive methods and with multinomial logistic regressions for the probability of visits and with negative binomial regressions for the number of visits, adjusted for sociodemographic covariates. The results showed a clear socioeconomic gradient for the probability of visits according to income and education: the higher the income and the higher the education, the more likely was a visit to a dentist–especially a private dentist–during the two-year period. Similar results were obtained for the number of visits. Higher socioeconomic status was less associated with public dentist visits. While those with the lowest income visited public dentists more frequently than private dentists, their overall visits fell below that of others. Adjusted estimates by occupation did not show a clear socioeconomic gradient. The socioeconomic inequality in dentist visits in a country having a universally covered public dental care scheme puts a challenge for decision makers in designing an equal dental health care system. Experimenting with lower co-payments is a possible option.

## Introduction

Poor dental and oral health has been associated with chronic diseases [1–4] and lower quality of life [1]. Access to and utilization of dental health care services is an important part of maintaining dental and oral health. Despite this, a significant share of residents in developed countries lack access or opt not to use dental health care services. On average, in the OECD countries in 2014, only 63% of those aged 15 or over reported visiting a dentist during the previous year [5]. However, partly due to differences in the coverage of dental care in the population, there is large variation between countries; for example, in the US, the share of the population who reported visiting a dentist in the previous year was 41% while in Ireland, it was 93% [5].

A social gradient has been reported for dental health: those having lower socioeconomic positions have poorer dental health [6–9]. Socioeconomic inequality in dental health may partly result from differential patterns of dental health care utilization. Previous studies, which are mostly based on survey data, have found that high-income earners have a significantly higher likelihood of utilizing dental care than low-income earners [10–12]. This result has been found in both adolescent [13] and elderly [14] populations. In addition to income, other socioeconomic indicators, such as higher educational attainment and employment status, have been found to have strong explanatory power in the variation of dental care utilization [10, 12, 15, 16]. This general finding that those with a lower socioeconomic status have both poorer health but utilize less health services is challenging from the perspective of organizing equal supply of dental health care for different population groups.

In many countries, dental care is offered by both public and private health care schemes. The Finnish dental health care provision is an example of a two-sector system where dental care provided through the public sector is universally covered for all residents and dental care provided in the private sector is free-market based [17]. Municipalities are responsible for providing basic public dental care (including dental checks and follow-up checks), which is financed through taxation, although small separate co-payments are usually included for the visit and the dental procedures. For example, in 2018, the ceiling for the basic co-payment for adults for a visit to a public dentist was 13.10 euros (around 15.51 USD) [18]. Specialized dental care is provided by both the hospital districts’ units and the municipalities’ health centers.

Because of the universal coverage in the public sector, the demand for public sector services has been excessive, which has showed up as long waiting times. For example, in October 2018, the proportion of patients with non-urgent appointments to a public dentist who had to wait for longer than three weeks was 45% [19]. The private sector has had a strong role in dental care provision. Waiting times are typically shorter in the private sector and the patient can choose his or her dentist freely. It is typical for private dentists to offer patients annual or biannual recalls while public dentists, due to lack of resources, mostly use regular recalls for children [20]. The average price for a basic oral examination in the private sector was 63 euros (70 USD, using the 2018 exchange rate) in 2018 [21]. The use of private dental care is also partly subsidized through the National Health Insurance (NHI) scheme. The NHI covers dental treatment and laboratory and X-ray examinations but excludes cosmetic procedures and prosthodontics. In 2018, the total NHI reimbursement rate for dental care was 14.2% [22]. The NHI coverage and reimbursement rate have been decreasing over the years, and starting from 2016, NHI reimbursement for a dental examination is available only every other calendar year unless the patient’s health status, verified by a dentist, requires it.

Differential utilization of public and private sector services among different socioeconomic groups can be a contributing factor to the polarization of the overall use of dental care. In Finland, even with the strong subsidization of health services, socioeconomic inequalities in utilization have remained pronounced in both regular health care [23–25] and dental care over the past few decades [24, 26]. Literature based on survey data on the use of dental services in universally covered versus market-based sectors has found that use of the private sector is highly associated with higher socioeconomic status, and, vice versa, use of the public sector is more likely among persons with a lower socioeconomic status [10, 27–30]. Similarly, in Finland, private sector use has been more likely in higher socioeconomic groups [10, 28, 30]. However, register-based evidence on the socioeconomic gradient of use and abstaining from use of dental services in these sectors individually or in combination is scarce. Additionally, not much is known on how well the different sectors compensate for each other within different socioeconomic classes.

The aim of this study was to examine how different socioeconomic factors are associated with the use of dental health care in the public and private sectors in Finland. Using comprehensive register data on the adult population of one Finnish city over a two-year period 2017–2018, we aimed to estimate the associations of education, occupational class, and income with using either public or private dental services or both, or not using dental health care at all. While education, occupational class and income are partly intertwined, they may also be related to the use of health care through different mechanisms. Education may correlate with the interest in maintaining good health and care demand, and relate to skills in navigating the health care system [31]. Occupational class may influence health-seeking behavior, for example, through occupational cultures. Income is directly related to the ability to pay for the services. In order to reduce inequalities in access and use of health care services, it is crucial to improve knowledge on how these socioeconomic factors influence the utilization of services organized in different health care sectors.

## Data and methods

### Data sources

Data were collected from several registers for the population aged 25 and over of the city of Oulu, Finland, for the period 2017–2018. Oulu, which is situated in Northern Finland, is the fifth largest city of the country, with approximately 200,000 residents. Data from different registers were linked using pseudonymized individual identifiers (for more detail, see Blomgren and Jäppinen 2020 [32]).

First, population data were derived from the registers of the Social Insurance Institution of Finland, including information on place of residence, age and sex. Second, register data on educational level and occupational class were retrieved from Statistics Finland. Third, annual-level information on taxable income was gathered from the Finnish Tax Administration. These data include both earned income and capital income. Fourth, register data on utilization of public dental health care services was obtained from the city of Oulu. This register contains information on the date of the visit, the type of service and contact, and the occupational class of the health care provider. Fifth, information on visits to private dental health care services was collected from the private health care reimbursement register, maintained by the Social Insurance Institution of Finland. This register includes all records of visits and procedures reimbursed by the NHI. We restricted the data to reimbursements of dentist visits.

We defined the study population as those who were residents of the city of Oulu during the whole period 2017–2018. This sample included 183,536 persons. We limited the study population to adults aged over 25 and non-students according to the information on their occupational class. The final sample included 118,397 persons.

### Outcome variables

In both public and private dental health care registers, a visit may contain several recorded procedures. As the primary interest was in visits, we distinguished individual visits by their date and patient identifier, that is, we set each patient to have a maximum of one visit per day. Additionally, concerning public dental care, we limited the data to contacts that were actual visits (as opposed to, for example, remote contacts by phone or email) using information on the contact type. We measured visits with a binary variable, i.e., whether an individual had no visits or at least one visit during the two-year period 2017–2018, and with the total number of visits during this time. We separately measured whether an individual had no dental health care visits, only public visits, only private visits, or visits to both private and public dental health care during the two-year period. We chose to use a two-year period for two reasons. First, it is typical for dentists to recommend booking a visit for regular checkups for every other year only. Second, in 2016, the NHI reimbursement was changed to cover visits in the private sector for only every other year.

### Covariates

Age was categorized into 10-year age bands: 25–34, 35–44, 45–54, 55–64, 65–74, and 75 and over. Additionally, we included a covariate for the sex of the resident. We measured socioeconomic background by education, occupational class, and total income. For age, occupational class and education, information from the beginning of 2017 was used. Total income was measured as the sum of earned income and capital income from the two-year period 2017–2018.

Based on highest obtained qualifications, the educational level was divided into groups of upper tertiary (over 15 years of education), lower tertiary (13–15 years), secondary (11–12 years), and up to basic level education. According to the classification of Statistics Finland [33], occupational class was divided into seven categories: upper-level non-manual employees (e.g., directors, managers, teachers, and physicians), lower-level non-manual employees (e.g., technicians, nurses, and police officers), manual workers (e.g., construction workers, transport workers, mechanics, and cleaners), self-employed (including owners of companies with salaried employees), unemployed, retired, and others (including unknown). We divided income into five different categories by quintiles.

Table 1 shows the descriptive statistics of the covariates. The gender ratio was quite equal in the study group. The mean age of the study population was 50.9 years. 44% of the population had a tertiary education and 39% had a secondary education. Of those who worked, lower-level non-manual employees was the most common occupational class (21%) but retirees were the largest group in the whole sample (30%). The mean total income from the two-year period in the study population was slightly under 71,000 euros (78,626 USD), with large variation between the quintiles. The mean income in the highest quintile and lowest quintile was, respectively, 151,410 and 21,510 euros (167,674 and 23,820 USD).

**Table 1:** Descriptive statistics of the covariates.

	N	%	Mean	Median
Sex				
Male	58,209	49.2		
Female	60,188	50.8		
Age			50.9	50.0
25–34	23,402	19.8	29.7	30.0
35–44	24,153	20.4	39.4	39.0
45–54	22,158	18.7	49.6	50.0
55–64	21,448	18.1	59.4	59.0
65–74	16,518	14.0	68.9	69.0
> 74	10,718	9.1	80.9	80.0
Education				
Upper tertiary	19,643	16.6		
Lower tertiary	32,095	27.1		
Secondary	46,366	39.2		
Basic	20,293	17.1		
Occupational class				
Upper-level non-manual employees	21,729	18.4		
Lower-level non-manual employees	25,175	21.3		
Manual worker	15,273	12.9		
Self-employed	5,571	4.7		
Unemployed	12,064	10.2		
Retired	35,485	30.0		
Other	3,100	2.6		
Income (thousand euros), quintiles			70.8	59.3
Quintile 5 (highest)	23,680	20.0	151.4	123.7
Quintile 4	23,679	20.0	80.4	79.6
Quintile 3	23,679	20.0	59.4	59.3
Quintile 2	23,679	20.0	41.2	41.2
Quintile1 (lowest)	23,680	20.0	21.5	21.9
Total	118,397	100.0		

Note: Study population: non-student (aged over 25) residents of Oulu in 2017–2018.

### Statistical analyses

We started by crudely examining the proportions of persons who had public and/or private dentist visits or no visits by their socioeconomic background during the two-year observation period. To further investigate how socioeconomic differences are related to dental health care use and to adjust for the confounding association between sex, age and other socioeconomic characteristics, we estimated a multinomial logit model by maximum likelihood [34]. The dependent variable was divided into four categories: no visits to a dentist, visited only public dentist, visited only private dentist, or visited both public and private dentists. The “no visits” category was chosen as the reference group. We reported the odds ratios (OR) of the estimates with their 95% confidence intervals.

Finally, to examine how socioeconomic differences are associated with the number of visits to dentists, we estimated negative binomial regression models by maximum likelihood estimation [34]. We chose to use negative binomial regression over Poisson regression because of overdispersion in the data, that is, the variance of visits was much larger than the mean of visits (S1 Fig). We estimated the model separately for three outcomes: number of total visits, number of visits to public dentists, and number of visits to private dentists. We reported the incidence rate ratios (IRR) of the estimates with their 95% confidence intervals. All the analyses were conducted using R version 4.0.4 [35].

### Ethics statement

This study was conducted using register-based data, and no human subjects were contacted in the data collection. The Finnish National Board on Research Integrity does not require an ethical review statement for studies conducted solely with register-based data [36]. We followed good scientific practice, data protection guidelines and ethical standards [36] in collecting and analyzing the data and in reporting the results. According to the General Data Protection Regulation of the EU (GDPR) [37] and the Finnish Data Protection Act [38], processing of personal data is permitted without informed consent for a task carried out in the public interest, such as scientific research. The data were accessed through permissions from the City of Oulu (date of permission 20 Jan 2021), the Social Insurance Institution of Finland (date of permission 11 Feb 2021), Statistics Finland (date of permission 22 Dec 2020) and the Finnish Tax Administration (date of permission 9 Feb 2021). Before distributing the data to researchers, the personal identifiers were pseudonymized by the data providers. When conducting the analyses, researchers never had access to the personal identifiers. Because of the sensitiveness of the pseudonymized data, legal restrictions prevent the public sharing of the data [38, 39]. The researchers do not have permission of the data providers for data sharing.

## Results

### Descriptive results

Table 2 shows the overall proportions of persons having dentist visits, and the average number of visits in the study population. On average, 38% had no dentist visits during the two-year period. The proportion of those who had only private visits (28%) was similar to those who had only public visits (29%). Having both public and private visits during the two-year period was uncommon (5%). On average, there were 2.1 visits per person in the study population during the two-year period, and they were rather evenly distributed between public and private dentists (Table 2).

**Table 2:** Overall visits to dentists.

	%	Mean
No visits	37.8	
Only public visits	29.1	
Only private visits	28.4	
Public and private visits	4.6	
Number of visits per person		2.14
Number of public visits per resident		1.06
Number of private visits per resident		1.08

Note: Study population: non-student (aged over 25) residents of Oulu, Finland, in 2017–2018.

**Fig 1:**
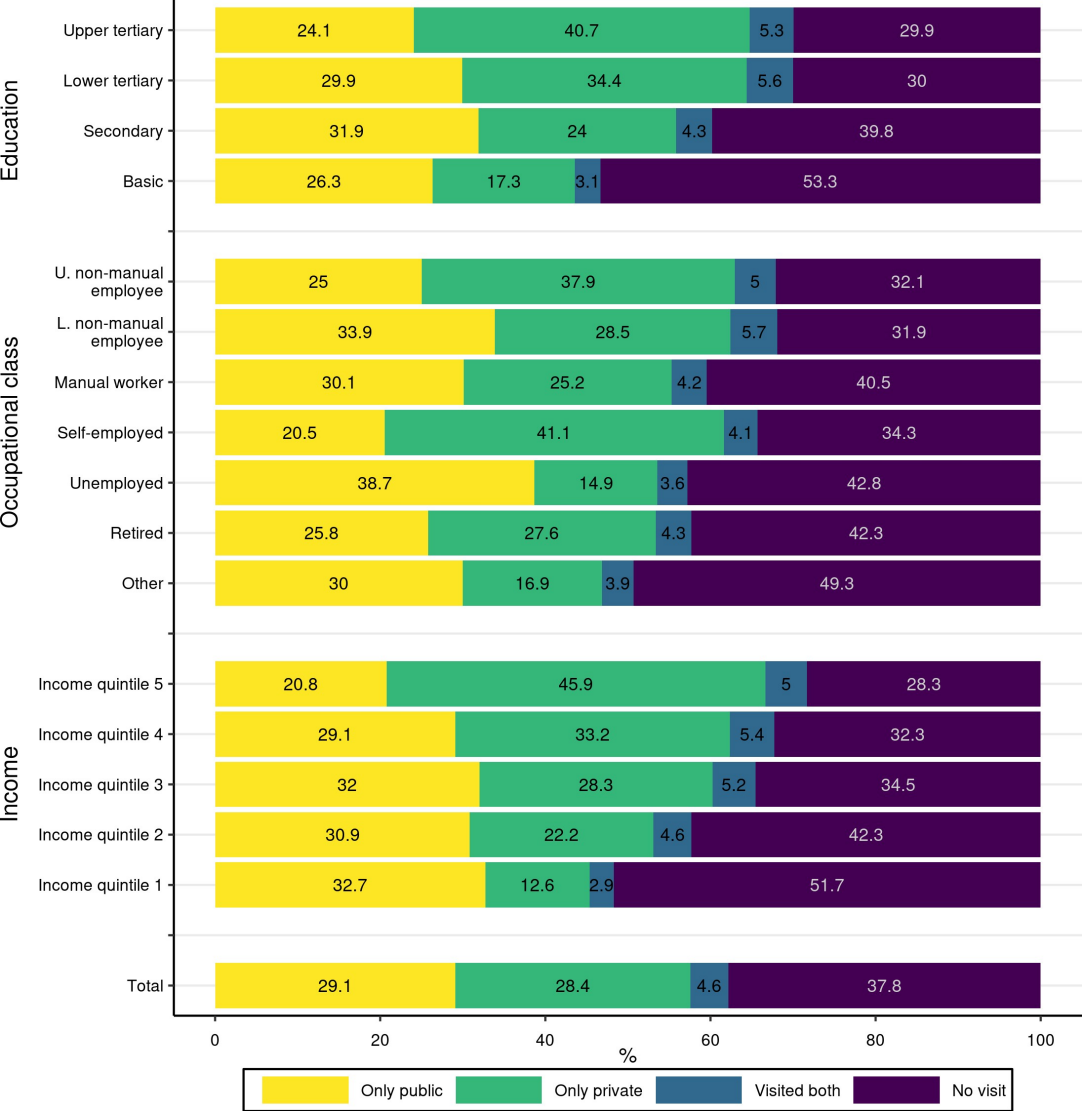
Proportions (%) of persons having public, private or no dentist visits during 2017–2018, by socioeconomic background. Study population: non-student (aged over 25) residents of Oulu, Finland, in 2017–2018 (N = 118,397).

Fig 1 shows the proportions of visits into different sectors according to socioeconomic variables.

The proportion of residents with a visit to a dentist and a visit to a private dentist was higher among those with higher educational attainment. The self-employed had the highest proportion of those who visited only private dentists (41%). Among manual workers and unemployed persons, the proportion of those with private dentist visits was lower than average. However, the unemployed had a higher proportion of those with public visits, which diminished the overall difference in the proportion of those who had no visits. The proportion of those who had no visits to dentists was lowest among the upper and lower non-manual workers.

The higher the level of income, the higher was the proportion of persons having private visits. The proportion of only private dentist visitors was 46% in the highest income quintile, while in the lowest income quantile it was only 13%. Those with higher income had also a higher overall proportion of those with a visit to a dentist. In the lowest quintile, half of the residents did not have a dentist visit over the two-year period while in the highest quintile, 28% did not visit a dentist.

### Multinomial logit regression for visiting probability

**Fig 2:**
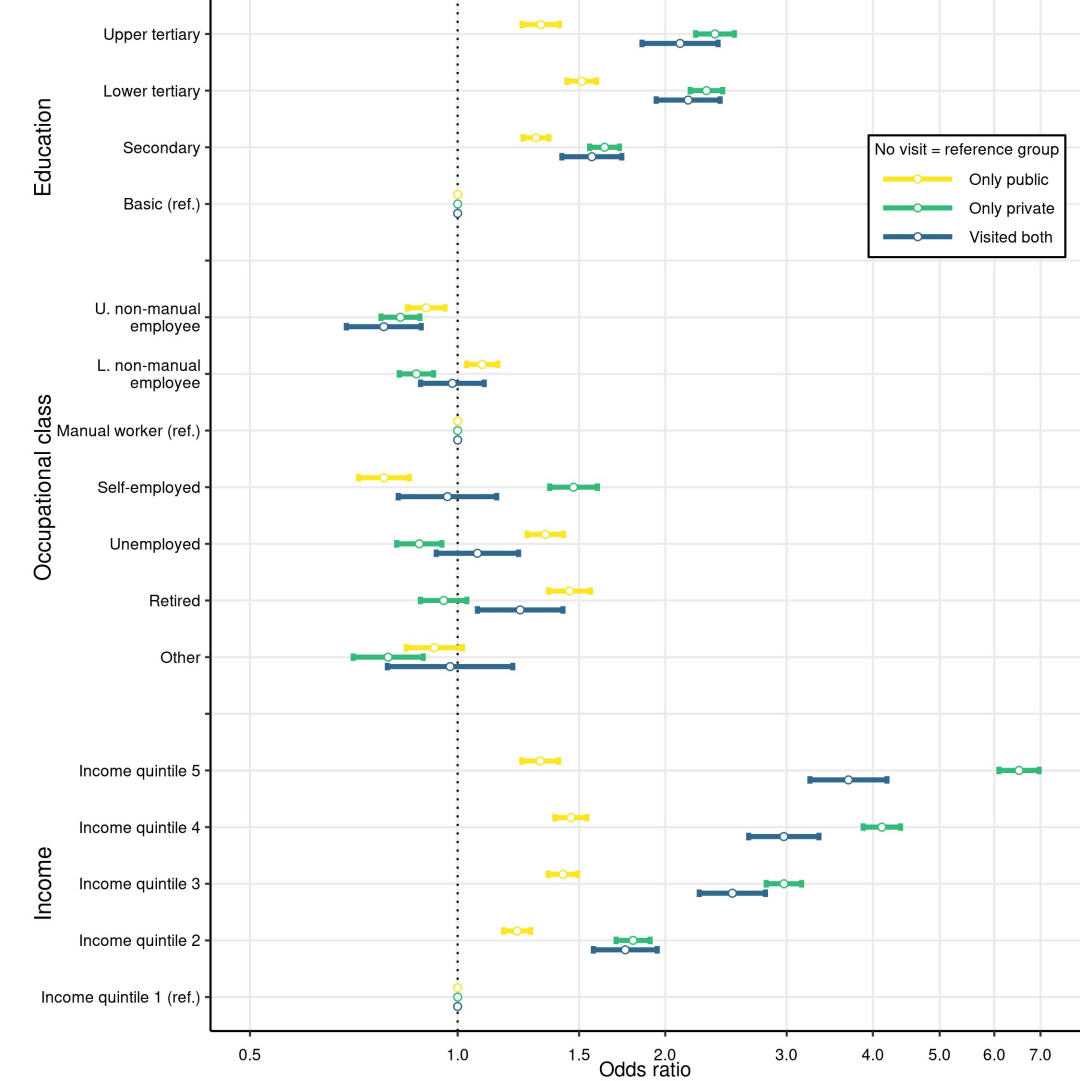
Multinomial logit odds ratios for visiting public, private, or public and private dentists, with their 95% confidence intervals. Study population: non-student (aged over 25) residents of Oulu, Finland, in 2017–2018 (N = 118,397). Other covariates included in the logit model but not shown in the figure are sex and age groups 25–34, 35–44, 45–54, 55–64, 65–74, and over 75. “No visit” is the reference category. The x-axis is scaled using log of base 10. The vertical dashed line is set at OR = 1.

Fig 2 shows the adjusted estimated ORs and their 95% confidence intervals from the multinomial logit model adjusted for sex, age and all socioeconomic variables simultaneously. Detailed estimates from the model are shown in S1 Table. For unadjusted estimates and estimates that are not adjusted for the income quantiles, see S2 Table. Higher education was associated with higher odds of having either a public or private dentist visit. In particular, those with upper or lower tertiary education had twice the odds of visiting only private dentists relative to those with basic education. The association between higher education and the OR for only public visits was lower than for a private visit.

When adjusted for other covariates, the likelihood of visiting only private dentists and higher occupational class was less notable. Compared to unadjusted results in S2 Table, for upper and lower-level non-manual workers, the higher likelihood of visiting private or public dentists vanished. Comparison of the estimates from the main model and unadjusted model shows that the educational attainment (rather than income) was the most relevant predictor of higher visiting likelihood among upper-level non-manual workers. In other words, it was the higher educational attainment among upper-level non-manual workers that explained the difference in the visits and not the occupational class itself. The share of residents that had attained at least a lower tertiary education was 89% among upper-level non-manual workers and 11% among manual workers. Relative to manual workers, only self-employed residents had significantly higher odds of visiting only private dentists. Unemployed and retired residents had the highest odds of visiting only public dentists.

In line with Fig 1, being in the higher income quintiles was associated with higher odds of visiting only private dentists. For those in the highest income quintile relative to the lowest income quintile, the odds of visiting only private dentists with respect to not visiting a dentist at all was over six times higher. In contrast, belonging to a higher income quintile increased the odds of visiting only public dentists much less: the OR relative to the lowest income quintile was below 1.5 in the other income quintiles. All the results above remained similar if estimated separately for the age groups 25–44, 45–64, and over 64 (results available from authors).

### Negative binomial regression for number of visits

**Fig 3:**
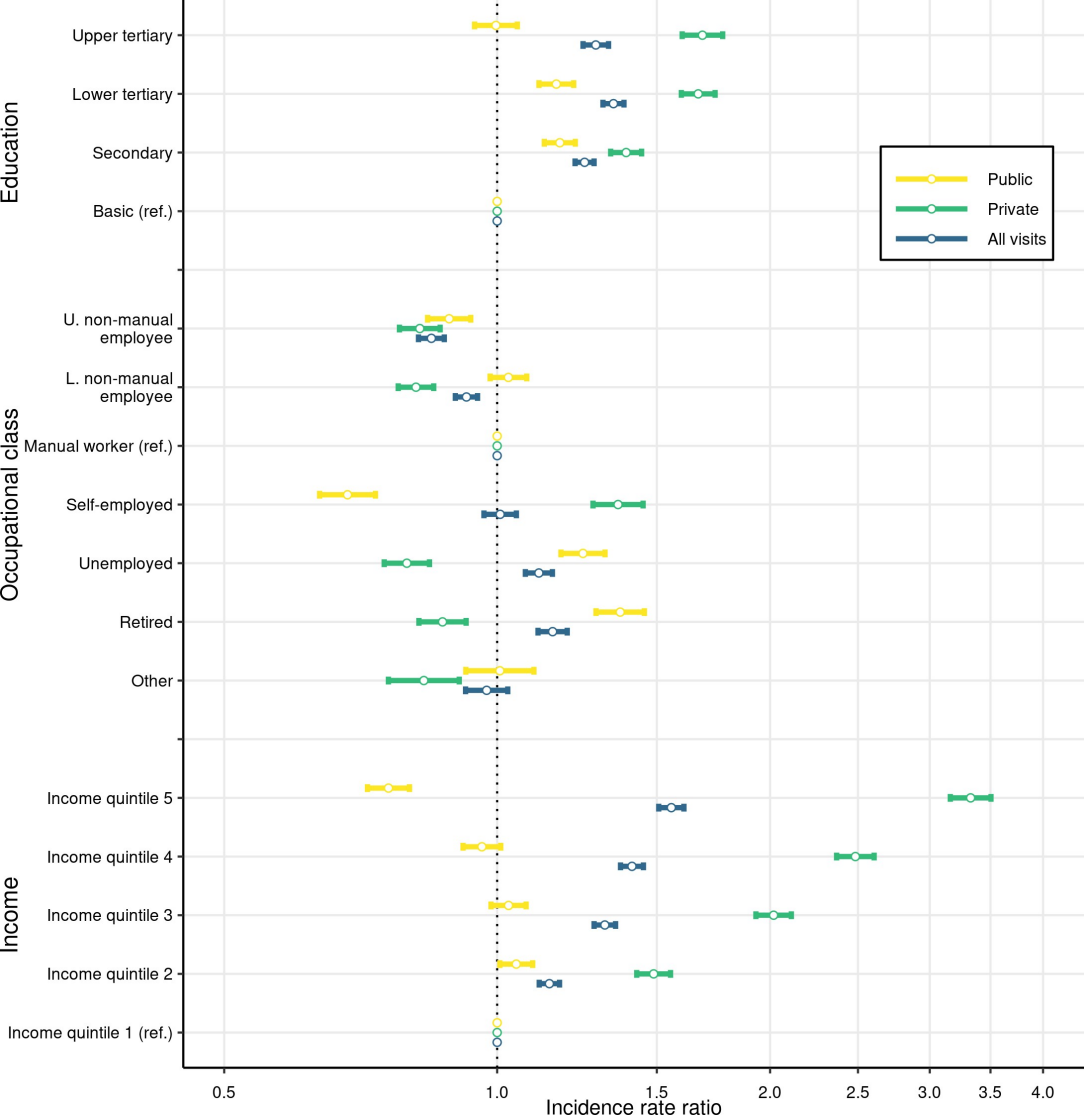
Negative binomial model incidence rate ratios for the number of public, private and all visits, with their 95% confidence intervals. Study population: non-student (aged over 25) residents of Oulu, Finland, in 2017–2018 (N = 118,397). Other covariates included in the model but not shown in the figure are sex and age groups 25–34, 35–44, 45–54, 55–64, 65–74, and over 75. The results for each dependent variable are estimated from separate regressions. The x-axis is scaled using log of base 10. The vertical dashed line is set at IRR = 1.

Fig 3 displays the estimated adjusted IRRs and their 95% confidence intervals from negative binomial models. Detailed estimates from the models are shown in S3 Table. Unadjusted estimates are shown in S4 Table. Higher educational attainment was associated with a higher expected number of visits to private dentists. Among those with upper tertiary education, the expected number of public visits did not differ from those with basic education. However, concerning all visits, the gradient was roughly similar to that for private visits only.

Similar to the results from the multinomial logit regression, self-employed residents had the highest expected number of private visits. While the unemployed and retired residents had lower expected number of private visits, their higher expected number of public visits compensated for this, and the overall expected number of visits was not below that of others. Interestingly, upper-level non-manual employees had a lower expected number of both private and public visits than manual workers. In contrast to the unadjusted estimates (S4 Table), and in line with the multinomial logit results, the overall expected number of visits among upper- and lower-level non-manual workers was lower than among the other occupational classes. Again, as shown in S4 Table, in the estimates that do not adjust for the income quintiles, the stronger association was caused by the higher educational attainment among upper- and lower non-manual workers.

Moving from the lowest income quintile to the highest income quintile increased the incidence rate of visiting private dentists by almost a factor of 3.5. Conversely, the expected number of visits to public dentists was not increased with income-level. The coincidental increase in the IRR of total visits and income seemed to result solely from private visits. All the estimated coefficients remained similar when estimated separately for younger and older residents (25–44, 45–64, and over 64-year-olds, results available from authors).

## Discussion

### Key results

In this paper, we examined the utilization of public and private dental health care by different socioeconomic factors using comprehensive register data for an adult population residing in a Finnish city over the period 2017–2018. We studied separately the utilization of only public and only private dentists, as well as abstaining from visiting dentists entirely. First, we provided crude descriptive evidence of dental health care visits within different educational, occupational, and income classes. Second, we estimated the adjusted association of these socioeconomic factors and the likelihood and the expected number of visits to public and private dentists.

The main finding is that the overall utilization of dental health care clearly differed between residents with higher and lower educational attainment and income. Especially high income was strongly associated with a higher likelihood of having at least one dental health care visit and with a higher total number of visits. The difference mainly stemmed from visits to private dentists, as higher socioeconomic status was less associated with the utilization of public dentists. For the lowest-income population, the number of visits to public dentists was not frequent enough to compensate for the lower number of private dentist visits, and the overall number of dentist visits fell below that of other groups. While those with a higher occupational class, i.e., upper or lower-level non-manual employees, had a higher than average probability of dentist visits in the unadjusted assessment, this association did not hold in models that simultaneously adjusted for the socioeconomic variables and other covariates.

### Implications of the results

Previous studies have shown that there is socioeconomic inequality in the use of dental care, so that the more privileged population groups utilize more of these services [10–12] despite the common finding that the disadvantaged population has poorer dental health [6–9]. The results of this study support these findings, especially the finding of pro-rich inequality in the utilization of dental services. Naturally, those with higher income have better possibility to pay the out-of-pocket expenses of dental care, which are substantial in the private sector but may be noteworthy also in public care. While income had the strongest association with dental care visits, also higher educational attainment was positively associated with higher utilization rates even when adjusting for income and other covariates. The result may reflect more health-conscious behavior and better understanding of the health care system among the higher educated [31]. This mechanism may partly explain the higher dental care utilization of the population with a higher socioeconomic status, that also tends to have better dental health and thus less need for dental care.

Previous studies that have been able to measure occupational status have mainly measured it by employment status [10, 12, 16, 27] and retirement status [11]. The main finding has been that being unemployed or retired is associated with less dental health care visits. Our adjusted estimates differ from these results: while unemployed and retired persons visited private dentists less frequently, their overall dental care visits did not fall behind that of other groups because of their more frequent public dentist visits. These results, which differ from those of other studies, may be partly explained by the highly subsidized Finnish public dental care, our possibility to comprehensively measure utilization in both sectors, and our use of register-based data instead of survey data.

The special contribution of our study to the literature was the ability to examine both public and private dentist visits by socioeconomic groups and to assess their joint contribution to the overall socioeconomic differences in utilization of dental care. The results showed that the observed socioeconomic inequality largely stems from a higher probability of using private dental care in the higher socioeconomic groups. Also, the overall utilization followed a similar pattern, albeit the distribution of the visits in the public sector partly mitigates the socioeconomic gradient. In terms of number of visits, the highest income group utilized more private sector services and less public sector services than the lower income groups. However, the number of public dentist visits was not large enough to close the gap in the difference in the overall number of visits. While previous studies have examined associations of socioeconomic characteristics with either choosing a public or private provider using survey data [10, 27–30], comprehensive joint examination of absence of visits and sector choice by socioeconomic status using register-based data has been lacking.

The Nordic health care system is based on a strong public sector that provides care for all residents with little or no charges. The public scheme aims to provide care that is affordable and accessible also to the disadvantaged population. The latest major reform in Finnish dental health care took place in 2001–2002, with the aim of improving provision and accessibility of dental care to the whole population. After the reform, all adults became entitled to subsidized public dental care and the NHI partial coverage was extended to private dental care for the total Finnish population [26]. Before the reform, mainly children and adolescents were entitled to public dental care, and only young age groups were entitled to NHI reimbursement for private dental care. However, studies have showed that, since the reform, pro-rich inequality in overall utilization of dental care has persisted [26, 30, 40]. Our results show that the issue is still topical: those in the lowest income group had the lowest likelihood and expected number of visits while those in the highest income group had the highest likelihood and expected number of visits.

How monetary-based subsidization schemes increase the utilization of dental services and dental health of the most disadvantaged population is still unclear. Some results from the literature suggest that extending dental insurance coverage in the general population is associated with increased use of these services [41–44]. However, the generalization of these results to the lowest income population and to the Finnish setting with already publicly covered dental care is likely to be poor. Besides low income, other potential reasons for low utilization rates include the lack of knowledge of the importance of dental care, long waiting times to public dentists, and the ability of the lowest-income patients to pay for the co-payments in public care. Also, a lower rate of recalls in the public sector than in the private sector [20] may contribute to infrequent visits among the lower income group. Future research should attempt to assess the role of these factors in explaining low use of dental care among the low-income population. Moreover, the causal effect of increased income on the utilization of dental services and the price-elasticity of public and private dental health care should be examined.

### Methodological considerations

We gathered population-wide register data from the city of Oulu and merged detailed individual-level demographic and socioeconomic characteristics as well as data on both public and private dental care visits from administrative registers. Register-based data are deemed to be highly reliable, have very little missing information and no loss to follow-up. In comparison to survey-based data, we were able to observe real utilization choices of different sectors and abstaining from dentist visits. Survey data may suffer from response bias that inflates the answers. For example, individuals may be ashamed of not visiting dentists and feel pressured to give socially desirable answers in surveys. Additionally, individuals may not remember correctly their past visits. It is common that the response rates in questionnaires are low and that those who answer the questionnaires can be systematically different from those who do not answer. These factors bias the results and the real magnitude of dental service utilization may remain unclear. The aforementioned challenges are largely tackled with register data.

A drawback that stems from the Finnish NHI scheme is the biannual coverage of visits. As the data contained only visits that were covered by the NHI scheme, some of the visits to private dentists may have been left unobserved. However, our use of a two-year follow-up period mitigates the problem. Nevertheless, the estimates regarding the number of private dentist visits should be considered as lower bound. Another drawback is that we did not observe dental health. Without observed dental health, inferences on whether higher utilization of dental services originates from poorer dental health and whether absence of dental service use is explained by good dental health cannot be directly made based on our data. However, previous literature strongly suggests that the disadvantaged population who consume the least dental care also has poorer dental health [6–9]. Assuming that these results are generalizable to our setting, that is, higher socioeconomic status and better dental health are positively associated, implications of need-based dental care with respect to socioeconomic status can be drawn.

The data were drawn from one geographical area, the city of Oulu, which is the fifth largest city in Finland. Even with the comprehensiveness of the data, it is useful to recognize the potential problems in the external validity of the results to the Finnish population. Naturally, the thickness and the size of the private dental health care market affect the supply side and the possibilities to choose to visit a private dentist. Likewise, the excess demand for public dentists and long waiting times can limit the choice to visit a public dentist. These two factors are quite similar in the largest cities in Finland, and thus, the results are likely to be very generalizable to other large cities. Instead, rural areas typically have low number of private dentists, which may hamper the generalizability of our results to these areas. In total, however, as 71% of the Finnish population lived in urban settlements in 2018 [45], the external validity of our results should be reasonably applicable to the aggregate Finnish population. Also, the population and living conditions in one city may vary from the country average. However, even though there are some differences as compared to the country as a whole, the city of Oulu does not differ in any systematic way from other large cities or from the average Finnish population. Thus, the results are well assumed to be generalizable to the whole of Finland [32]. As our results showed a similar socioeconomic gradient in dental care utilization as many previous studies from other settings, our results should also be applicable to other countries that have a tax-based public dental care sector and market-based private sector.

Because of the lack of an experimental study design, our estimated coefficients do not have a causal interpretation. Unobserved confounders may simultaneously affect an individual’s educational attainment, occupation, income, and utilization of dental services. For example, more health-conscious individuals who utilize dental services regularly may also aim for higher educational attainment and higher paid jobs. Thus, the estimated associations may be partly explained by uncontrolled confounders. Also, individuals with higher income may more frequently purchase voluntary private insurance, which may be associated with more frequent use of private services. However, in Finland, private insurance does not typically cover dental care, and therefore this factor is unlikely to explain our results.

## Conclusions and policy implications

Higher income and education was associated with higher a probability and number of visits, especially to private dentists. Visits to the public sector mitigated some of the gap in the visits between high-income and lower-income groups. Those in the lowest income and education group had the highest absenteeism rate.

The Finnish health care system is in the midst of a major structural reform where the goal is to increase the efficiency of health care provision and to reduce inequalities in access and use of services. The plan is to provide more centralized health care by transferring the responsibility from municipalities to larger geographical areas. According to the plan, also the financing of health services is shifted to these larger areas. Putting more resources to experimenting with lower co-payments and automatic recall systems in the low-income population are potential directions in reducing inequality. In order for policy makers to comprehend the present state of the use of health services and to design an equal health care system, it is crucial to have up-to-date information on the use of different sectors. Evaluation of various reforms, experiments, and system-specific differences using registers should be regular.

## Supporting information

S1 Fig Distribution of visits. Study population: non-student (aged over 25) residents of Oulu in 2017–2018 (N = 118,397).
(TIF)

S1 Table Multinomial logit odds ratios. (DOCX)

S2 Table Multinomial logit odds ratios for unadjusted and intermediary model. (DOCX)

S3 Table Negative binomial model incidence rate ratios. (DOCX)

S4 Table Negative binomial model incidence rate ratios for unadjusted and intermediary model. (DOCX)
