# Supplementary figures and images for: Socioeconomic differences in utilization of public and private dental care in Finland: Register-based evidence on a population aged 25 and over
Source: PLoS One. 2021 Aug 4;16(8):e0255126. doi: 10.1371/journal.pone.0255126 (PMC8336838; doi:10.1371/journal.pone.0255126)

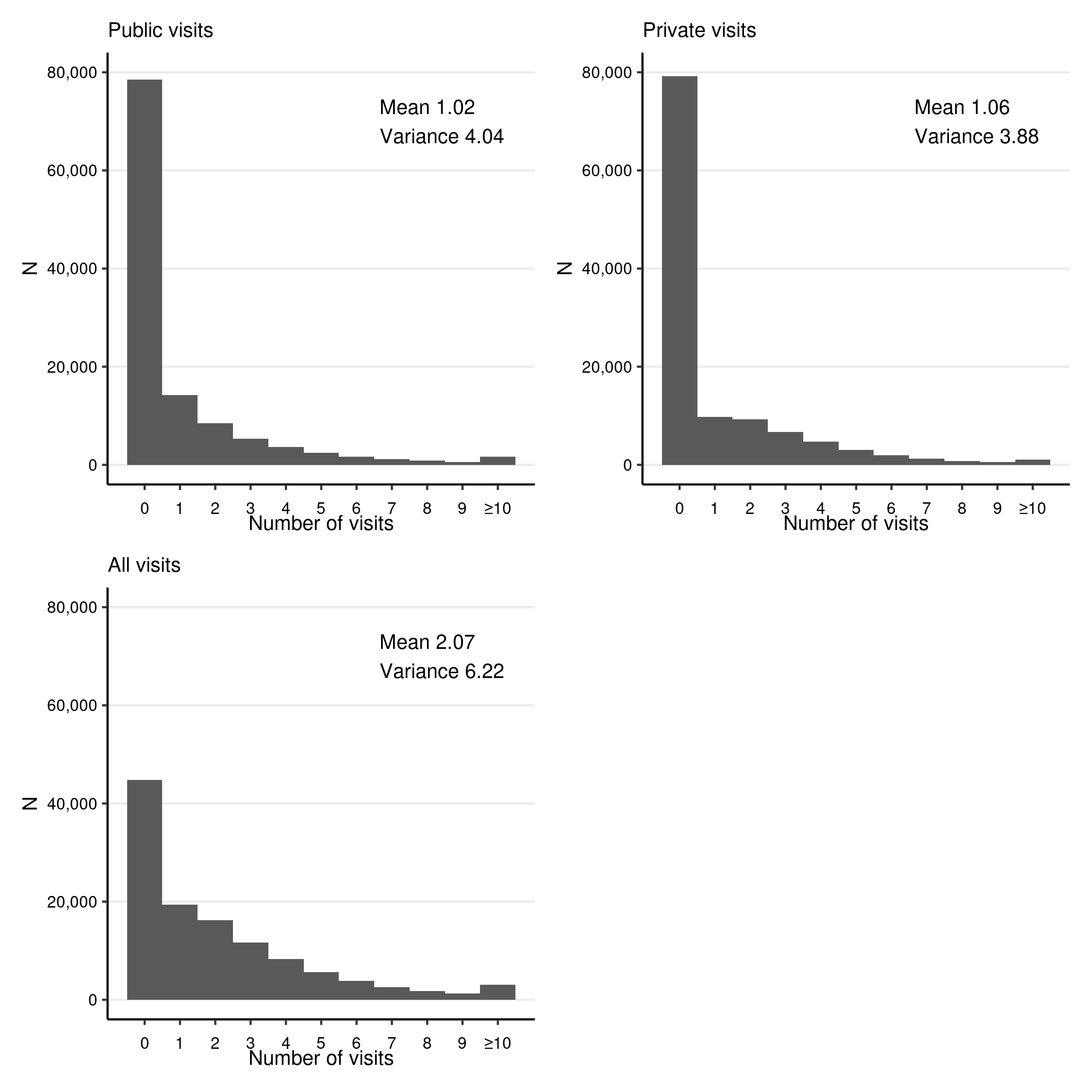

Supplement: S1 Fig — Study population: non-student (aged over 25) residents of Oulu in 2017–2018 (N = 118,397). (TIF) [file pone.0255126.s001.tif]
