# Supplementary material for: Socioeconomic differences in utilization of public and private dental care in Finland: Register-based evidence on a population aged 25 and over
Source: PLoS One. 2021 Aug 4;16(8):e0255126. doi: 10.1371/journal.pone.0255126 (PMC8336838; doi:10.1371/journal.pone.0255126)
Supplement: S1 Table — (DOCX) [file pone.0255126.s002.docx]

**S1 Table. Multinomial logit odds ratios.**

|  | **Only public** | | **Only private** | | **Visited both** | |
| --- | --- | --- | --- | --- | --- | --- |
|  | **OR** | **CI** | **OR** | **CI** | **OR** | **CI** |
| Intercept | 0.35 | (0.32, 0.37) | 0.06 | (0.05, 0.06) | 0.02 | (0.01, 0.02) |
| Education | | | | | | |
| Upper tertiary | 1.32 | (1.24, 1.41) | 2.36 | (2.21, 2.52) | 2.10 | (1.85, 2.39) |
| Lower tertiary | 1.51 | (1.44, 1.59) | 2.30 | (2.17, 2.42) | 2.16 | (1.94, 2.40) |
| Secondary | 1.30 | (1.24, 1.36) | 1.63 | (1.55, 1.72) | 1.57 | (1.42, 1.73) |
| Basic (ref.) |  |  |  |  |  |  |
| Occupational class | | | | | | |
| U. non-manual employee | 0.90 | (0.85, 0.96) | 0.83 | (0.77, 0.88) | 0.78 | (0.69, 0.89) |
| L. non-manual employee | 1.09 | (1.03, 1.14) | 0.87 | (0.82, 0.92) | 0.98 | (0.88, 1.09) |
| Manual worker (ref.) |  |  |  |  |  |  |
| Self-employed | 0.78 | (0.72, 0.85) | 1.47 | (1.36, 1.59) | 0.97 | (0.82, 1.14) |
| Unemployed | 1.34 | (1.26, 1.42) | 0.88 | (0.82, 0.95) | 1.07 | (0.93, 1.23) |
| Retired | 1.45 | (1.35, 1.56) | 0.95 | (0.88, 1.03) | 1.23 | (1.07, 1.42) |
| Other | 0.93 | (0.84, 1.02) | 0.79 | (0.71, 0.89) | 0.98 | (0.79, 1.20) |
| Income quantile | | | | | | |
| Quantile 5 | 1.32 | (1.24, 1.40) | 6.52 | (6.09, 6.97) | 3.69 | (3.24, 4.19) |
| Quantile 4 | 1.46 | (1.38, 1.54) | 4.12 | (3.87, 4.39) | 2.97 | (2.64, 3.34) |
| Quantile 3 | 1.42 | (1.35, 1.49) | 2.97 | (2.80, 3.15) | 2.50 | (2.24, 2.79) |
| Quantile 2 | 1.22 | (1.17, 1.28) | 1.80 | (1.70, 1.90) | 1.75 | (1.57, 1.95) |
| Quantile 1 (ref.) |  |  |  |  |  |  |
| Sex | | | | | | |
| Male (ref.) |  |  |  |  |  |  |
| Female | 1.60 | (1.55, 1.65) | 1.81 | (1.75, 1.87) | 2.33 | (2.18, 2.48) |
| Age group | | | | | | |
| 25-34 (ref.) |  |  |  |  |  |  |
| 35-44 | 1.06 | (1.02, 1.11) | 1.26 | (1.19, 1.32) | 1.08 | (0.99, 1.19) |
| 45-54 | 1.34 | (1.28, 1.40) | 2.22 | (2.11, 2.34) | 1.63 | (1.48, 1.79) |
| 55-64 | 1.14 | (1.09, 1.20) | 4.13 | (3.92, 4.37) | 2.30 | (2.08, 2.53) |
| 65-74 | 0.65 | (0.61, 0.70) | 3.25 | (3.00, 3.53) | 1.38 | (1.19, 1.60) |
| > 74 | 0.40 | (0.37, 0.43) | 1.92 | (1.75, 2.10) | 0.79 | (0.66, 0.93) |

Notes: The table shows the estimated odds rations (OR) and 95% confidence intervals (CI). Study population: non-student (aged over 25) residents of Oulu in 2017–2018 (N = 118,397).
