# Supplementary material for: Socioeconomic differences in utilization of public and private dental care in Finland: Register-based evidence on a population aged 25 and over
Source: PLoS One. 2021 Aug 4;16(8):e0255126. doi: 10.1371/journal.pone.0255126 (PMC8336838; doi:10.1371/journal.pone.0255126)
Supplement: S2 Table — (DOCX) [file pone.0255126.s003.docx]

**S2 Table. Multinomial logit odds ratios for unadjusted and intermediary model.**

|  | **Only public** | | | | **Only private** | | | | **Visited both** | | | |
| --- | --- | --- | --- | --- | --- | --- | --- | --- | --- | --- | --- | --- |
|  | **Unadj.** | | **Intermediary** | | **Unadj.** | | **Intermediary** | | **Unadj.** | | **Intermediary** | |
|  | **OR** | **CI** | **OR** | **CI** | **OR** | **CI** | **OR** | **CI** | **OR** | **CI** | **OR** | **CI** |
| Education | | | | | | | | | | | | |
| Upper tertiary | 1.63 | (1.55, 1.71) | 1.45 | (1.37, 1.54) | 4.20 | (3.99, 4.42) | 4.23 | (3.98, 4.50) | 3.11 | (2.80, 3.45) | 3.08 | (2.73, 3.48) |
| Lower tertiary | 2.02 | (1.93, 2.11) | 1.66 | (1.58, 1.74) | 3.54 | (3.38, 3.71) | 3.39 | (3.22, 3.57) | 3.27 | (2.98, 3.60) | 2.86 | (2.58, 3.17) |
| Secondary | 1.62 | (1.56, 1.69) | 1.36 | (1.30, 1.42) | 1.86 | (1.78, 1.95) | 1.89 | (1.80, 1.99) | 1.90 | (1.74, 2.09) | 1.76 | (1.60, 1.95) |
| Basic (ref.) |  |  |  |  |  |  |  |  |  |  |  |  |
| Occupational class | | | | | | | | | | | | |
| U. non-manual employee | 1.05 | (1.00, 1.10) | 0.86 | (0.81, 0.91) | 1.90 | (1.80, 2.00) | 0.94 | (0.88, 1.00) | 1.48 | (1.33, 1.64) | 0.82 | (0.73, 0.93) |
| L. non-manual employee | 1.43 | (1.36, 1.50) | 1.08 | (1.03, 1.14) | 1.44 | (1.36, 1.51) | 0.91 | (0.86, 0.96) | 1.71 | (1.55, 1.89) | 1.01 | (0.91, 1.12) |
| Manual worker (ref.) |  |  |  |  |  |  |  |  |  |  |  |  |
| Self-employed | 0.80 | (0.74, 0.87) | 0.72 | (0.66, 0.78) | 1.92 | (1.79, 2.07) | 1.31 | (1.21, 1.41) | 1.14 | (0.97, 1.34) | 0.85 | (0.72, 0.99) |
| Unemployed | 1.22 | (1.15, 1.28) | 1.11 | (1.05, 1.18) | 0.56 | (0.52, 0.60) | 0.43 | (0.40, 0.46) | 0.81 | (0.71, 0.91) | 0.63 | (0.56, 0.72) |
| Retired | 0.82 | (0.78, 0.86) | 1.21 | (1.14, 1.30) | 1.05 | (1.00, 1.10) | 0.50 | (0.47, 0.54) | 0.98 | (0.89, 1.08) | 0.75 | (0.66, 0.86) |
| Other | 0.82 | (0.75, 0.89) | 0.79 | (0.72, 0.86) | 0.55 | (0.49, 0.61) | 0.50 | (0.44, 0.55) | 0.75 | (0.61, 0.92) | 0.66 | (0.54, 0.81) |
| Income quantile | | | | | | | | | | | | |
| Quantile 5 | 1.16 | (1.11, 1.21) |  |  | 6.74 | (6.40, 7.10) |  |  | 3.21 | (2.90, 3.55) |  |  |
| Quantile 4 | 1.43 | (1.37, 1.50) |  |  | 4.35 | (4.12, 4.58) |  |  | 3.01 | (2.72, 3.33) |  |  |
| Quantile 3 | 1.43 | (1.37, 1.50) |  |  | 3.35 | (3.18, 3.54) |  |  | 2.74 | (2.48, 3.03) |  |  |
| Quantile 2 | 1.12 | (1.07, 1.17) |  |  | 2.11 | (2.00, 2.23) |  |  | 1.87 | (1.69, 2.08) |  |  |
| Quantile 1 (ref.) |  |  |  |  |  |  |  |  |  |  |  |  |
| Sex | | | | | | | | | | | | |
| Male (ref.) |  |  |  |  |  |  |  |  |  |  |  |  |
| Female | 1.53 | (1.49, 1.57) | 1.55 | (1.50, 1.59) | 1.38 | (1.34, 1.42) | 1.34 | (1.30, 1.38) | 2.00 | (1.89, 2.12) | 1.93 | (1.81, 2.05) |
| Age group | | | | | | | | | | | | |
| 25-34 (ref.) |  |  |  |  |  |  |  |  |  |  |  |  |
| 35-44 | 1.08 | (1.04, 1.13) | 1.08 | (1.04, 1.13) | 1.72 | (1.63, 1.80) | 1.49 | (1.42, 1.57) | 1.29 | (1.18, 1.42) | 1.20 | (1.10, 1.32) |
| 45-54 | 1.38 | (1.32, 1.44) | 1.36 | (1.30, 1.42) | 2.96 | (2.82, 3.11) | 2.74 | (2.60, 2.88) | 1.93 | (1.76, 2.11) | 1.85 | (1.68, 2.03) |
| 55-64 | 1.26 | (1.20, 1.32) | 1.19 | (1.13, 1.25) | 4.22 | (4.02, 4.44) | 5.08 | (4.82, 5.36) | 2.44 | (2.23, 2.67) | 2.65 | (2.41, 2.92) |
| 65-74 | 0.83 | (0.79, 0.87) | 0.72 | (0.67, 0.78) | 2.47 | (2.35, 2.60) | 4.86 | (4.49, 5.26) | 1.43 | (1.30, 1.59) | 1.88 | (1.63, 2.18) |
| > 74 | 0.49 | (0.46, 0.52) | 0.44 | (0.41, 0.48) | 1.21 | (1.14, 1.28) | 2.93 | (2.68, 3.19) | 0.73 | (0.65, 0.83) | 1.10 | (0.93, 1.30) |

Notes: The table shows the estimated odds ratios (OR) and 95% confidence intervals (CI). In the unadjusted model, each covariate group is separately and one at a time estimated on the outcome. The intermediary model is the same as the full model except the covariate for income quantile is omitted. Study population: non-student (aged over 25) residents of Oulu in 2017-2018 (N = 118,397).
