# Supplementary material for: Socioeconomic differences in utilization of public and private dental care in Finland: Register-based evidence on a population aged 25 and over
Source: PLoS One. 2021 Aug 4;16(8):e0255126. doi: 10.1371/journal.pone.0255126 (PMC8336838; doi:10.1371/journal.pone.0255126)
Supplement: S3 Table — (DOCX) [file pone.0255126.s004.docx]

**S3 Table. Negative binomial model incidence rate ratios.**

|  | **Public** | | **Private** | | **All visits** | |
| --- | --- | --- | --- | --- | --- | --- |
|  | **IRR** | **CI** | **IRR** | **CI** | **IRR** | **CI** |
| Intercept | 0.88 | (0.83, 0.93) | 0.17 | (0.16, 0.19) | 0.94 | (0.91, 0.98) |
| Education | | | | | | |
| Upper tertiary | 1.00 | (0.94, 1.05) | 1.68 | (1.60, 1.77) | 1.28 | (1.24, 1.33) |
| Lower tertiary | 1.16 | (1.11, 1.21) | 1.67 | (1.60, 1.74) | 1.34 | (1.31, 1.38) |
| Secondary | 1.17 | (1.13, 1.22) | 1.39 | (1.33, 1.44) | 1.25 | (1.22, 1.28) |
| Basic (ref.) |  |  |  |  |  |  |
| Occupational class | | | | | | |
| U. non-manual employee | 0.89 | (0.84, 0.94) | 0.82 | (0.78, 0.87) | 0.85 | (0.82, 0.87) |
| L. non-manual employee | 1.03 | (0.98, 1.08) | 0.81 | (0.78, 0.85) | 0.92 | (0.90, 0.95) |
| Manual worker (ref.) |  |  |  |  |  |  |
| Self-employed | 0.68 | (0.64, 0.73) | 1.36 | (1.28, 1.45) | 1.01 | (0.97, 1.05) |
| Unemployed | 1.24 | (1.18, 1.32) | 0.80 | (0.75, 0.84) | 1.11 | (1.07, 1.15) |
| Retired | 1.37 | (1.29, 1.45) | 0.87 | (0.82, 0.92) | 1.15 | (1.11, 1.19) |
| Other | 1.01 | (0.92, 1.10) | 0.83 | (0.76, 0.91) | 0.97 | (0.92, 1.03) |
| Income quantile | | | | | | |
| Quantile 5 | 0.76 | (0.72, 0.80) | 3.33 | (3.16, 3.50) | 1.56 | (1.51, 1.61) |
| Quantile 4 | 0.96 | (0.92, 1.01) | 2.48 | (2.37, 2.60) | 1.41 | (1.37, 1.45) |
| Quantile 3 | 1.03 | (0.98, 1.08) | 2.02 | (1.93, 2.11) | 1.31 | (1.28, 1.35) |
| Quantile 2 | 1.05 | (1.01, 1.09) | 1.49 | (1.43, 1.55) | 1.14 | (1.11, 1.17) |
| Quantile 1 (ref.) |  |  |  |  |  |  |
| Sex | | | | | | |
| Male (ref.) |  |  |  |  |  |  |
| Female | 1.20 | (1.17, 1.23) | 1.34 | (1.30, 1.37) | 1.27 | (1.25, 1.30) |
| Age group | | | | | | |
| 25-34 (ref.) |  |  |  |  |  |  |
| 35-44 | 1.07 | (1.03, 1.12) | 1.24 | (1.19, 1.29) | 1.09 | (1.06, 1.12) |
| 45-54 | 1.20 | (1.15, 1.25) | 1.91 | (1.83, 1.99) | 1.42 | (1.38, 1.45) |
| 55-64 | 0.95 | (0.91, 0.99) | 3.09 | (2.96, 3.22) | 1.62 | (1.58, 1.66) |
| 65-74 | 0.66 | (0.62, 0.71) | 3.02 | (2.83, 3.21) | 1.27 | (1.22, 1.32) |
| > 74 | 0.52 | (0.48, 0.56) | 2.36 | (2.20, 2.53) | 0.98 | (0.93, 1.02) |

Notes: The table shows the estimated incidence rate ratios (IRR) and 95% confidence intervals (CI). Study population: non-student (aged over 25) residents of Oulu in 2017–2018 (N = 118,397).
