# Supplementary material for: Socioeconomic differences in utilization of public and private dental care in Finland: Register-based evidence on a population aged 25 and over
Source: PLoS One. 2021 Aug 4;16(8):e0255126. doi: 10.1371/journal.pone.0255126 (PMC8336838; doi:10.1371/journal.pone.0255126)
Supplement: S4 Table — (DOCX) [file pone.0255126.s005.docx]

**S4 Table. Negative binomial model incidence rate ratios for unadjusted and intermediary model.**

|  | **Public** | | | | **Private** | | | | **All visits** | | | |
| --- | --- | --- | --- | --- | --- | --- | --- | --- | --- | --- | --- | --- |
|  | **Unadj.** | | **Intermediary** | | **Unadj.** | | **Intermediary** | | **Unadj.** | | **Intermediary** | |
|  | **IRR** | **CI** | **IRR** | **CI** | **IRR** | **CI** | **IRR** | **CI** | **IRR** | **CI** | **IRR** | **CI** |
| Education | | | | | | | | | | | | |
| Upper tertiary | 0.85 | (0.81, 0.89) | 0.92 | (0.87, 0.96) | 2.24 | (2.15, 2.34) | 2.45 | (2.33, 2.57) | 1.42 | (1.38, 1.46) | 1.49 | (1.45, 1.54) |
| Lower tertiary | 1.15 | (1.11, 1.20) | 1.13 | (1.08, 1.18) | 2.00 | (1.92, 2.08) | 2.14 | (2.05, 2.23) | 1.50 | (1.46, 1.53) | 1.49 | (1.45, 1.53) |
| Secondary | 1.25 | (1.21, 1.30) | 1.17 | (1.13, 1.22) | 1.37 | (1.32, 1.43) | 1.54 | (1.48, 1.60) | 1.30 | (1.27, 1.33) | 1.30 | (1.27, 1.33) |
| Basic (ref.) |  |  |  |  |  |  |  |  |  |  |  |  |
| Occupational class | | | | | | | | | | | | |
| U. non-manual employee | 0.76 | (0.72, 0.79) | 0.82 | (0.77, 0.86) | 1.39 | (1.33, 1.46) | 0.88 | (0.83, 0.92) | 1.05 | (1.02, 1.08) | 0.86 | (0.84, 0.89) |
| L. non-manual employee | 1.11 | (1.06, 1.16) | 1.02 | (0.97, 1.07) | 1.10 | (1.05, 1.15) | 0.82 | (0.78, 0.86) | 1.10 | (1.07, 1.13) | 0.93 | (0.90, 0.96) |
| Manual worker (ref.) |  |  |  |  |  |  |  |  |  |  |  |  |
| Self-employed | 0.66 | (0.61, 0.70) | 0.67 | (0.62, 0.71) | 1.64 | (1.54, 1.76) | 1.27 | (1.19, 1.36) | 1.11 | (1.07, 1.16) | 0.98 | (0.94, 1.02) |
| Unemployed | 1.30 | (1.23, 1.37) | 1.29 | (1.22, 1.36) | 0.60 | (0.57, 0.63) | 0.51 | (0.48, 0.54) | 0.97 | (0.94, 1.01) | 0.93 | (0.90, 0.96) |
| Retired | 0.96 | (0.92, 1.00) | 1.43 | (1.35, 1.51) | 1.21 | (1.16, 1.27) | 0.59 | (0.55, 0.62) | 1.08 | (1.05, 1.10) | 0.97 | (0.94, 1.01) |
| Other | 1.00 | (0.92, 1.09) | 1.02 | (0.93, 1.11) | 0.69 | (0.63, 0.76) | 0.64 | (0.59, 0.70) | 0.86 | (0.81, 0.90) | 0.86 | (0.82, 0.91) |
| Income quantile | | | | | | | | | | | | |
| Quantile 5 | 0.61 | (0.58, 0.63) |  |  | 3.50 | (3.36, 3.64) |  |  | 1.42 | (1.38, 1.45) |  |  |
| Quantile 4 | 0.86 | (0.83, 0.90) |  |  | 2.61 | (2.50, 2.72) |  |  | 1.35 | (1.32, 1.38) |  |  |
| Quantile 3 | 0.95 | (0.92, 0.99) |  |  | 2.26 | (2.17, 2.35) |  |  | 1.32 | (1.29, 1.35) |  |  |
| Quantile 2 | 0.95 | (0.91, 0.99) |  |  | 1.77 | (1.70, 1.85) |  |  | 1.18 | (1.15, 1.21) |  |  |
| Quantile 1 (ref.) |  |  |  |  |  |  |  |  |  |  |  |  |
| Sex | | | | | | | | | | | | |
| Male (ref.) |  |  |  |  |  |  |  |  |  |  |  |  |
| Female | 1.28 | (1.25, 1.31) | 1.27 | (1.24, 1.30) | 1.14 | (1.11, 1.16) | 1.09 | (1.06, 1.12) | 1.20 | (1.19, 1.22) | 1.19 | (1.17, 1.20) |
| Age group | | | | | | | | | | | | |
| 25-34 (ref.) |  |  |  |  |  |  |  |  |  |  |  |  |
| 35-44 | 0.99 | (0.95, 1.03) | 1.04 | (1.00, 1.08) | 1.51 | (1.45, 1.57) | 1.37 | (1.31, 1.42) | 1.15 | (1.13, 1.18) | 1.13 | (1.10, 1.16) |
| 45-54 | 1.13 | (1.08, 1.17) | 1.15 | (1.11, 1.20) | 2.37 | (2.28, 2.47) | 2.17 | (2.08, 2.26) | 1.53 | (1.49, 1.56) | 1.49 | (1.45, 1.52) |
| 55-64 | 1.01 | (0.97, 1.05) | 0.92 | (0.88, 0.96) | 3.33 | (3.20, 3.47) | 3.55 | (3.41, 3.70) | 1.75 | (1.71, 1.79) | 1.71 | (1.67, 1.76) |
| 65-74 | 0.88 | (0.84, 0.92) | 0.64 | (0.60, 0.68) | 2.68 | (2.57, 2.81) | 3.90 | (3.67, 4.15) | 1.46 | (1.42, 1.50) | 1.43 | (1.37, 1.48) |
| > 74 | 0.69 | (0.65, 0.72) | 0.50 | (0.47, 0.54) | 1.89 | (1.79, 1.98) | 3.09 | (2.88, 3.31) | 1.07 | (1.04, 1.10) | 1.10 | (1.06, 1.15) |

Notes: The table shows the estimated incidence rate ratios (IRR) and 95% confidence intervals (CI). In the unadjusted model, each covariate group is separately and one at a time estimated on the outcomes. The intermediary model is the same as the full model except the covariate for income quantile is omitted. Study population: non-student (aged over 25) residents of Oulu in 2017-2018 (N = 118,397).
